# Dietary habits among solid organ transplant recipients: results from a single-center study in Poland

**DOI:** 10.3389/fnut.2026.1685314

**Published:** 2026-05-20

**Authors:** Olga M. Rostkowska, Marta J. Gonciarz, Bartosz Olkowski, Dorota Miszewska-Szyszkowska, Olga Kozińska-Przybył, Daniel Śliż, Tomasz Warężak, Magdalena Durlik, Zuzanna Marczak

**Affiliations:** 1 Department of Transplantation, Immunology, Nephrology and Internal Diseases, Medical University of Warsaw, Warsaw, Poland; 2 Department of Diabetology and Internal Diseases, Medical University of Warsaw, Warsaw, Poland; 3 Students’ Scientific Group of Nephrology and Transplantation by the Department of Transplantation, Immunology, Nephrology and Internal Diseases, Medical University of Warsaw, Warsaw, Poland; 4 3rd Department of Internal Diseases and Cardiology, Medical University of Warsaw, Warsaw, Poland; 5 Engineering Research Institute, Universidad Cooperativa de Colombia, Medellín, Colombia

**Keywords:** BMI, eating habits, kidney transplantation, lifestyle medicine, Poland, post-transplant care, solid organ transplantation, survey

## Abstract

**Introduction:**

Solid organ transplant recipients are at risk of elevated BMI to which eating habits can contribute. Methods: The research tool was a questionnaire assessing dietary behaviors, distributed among patients in one transplant centre in Warsaw, Poland. Patients were asked about food frequency, types, and dietary changes after transplantation.

**Results:**

We included 205 respondents, 44.9% females. Mean age of all participants was 48.38 ± 13.68, median BMI = 24.86 kg/m^2^. Dietary behavior analysis revealed that patients who frequently consumed processed meats also tended to consume more animal fats (*ρ* = 0.41, *p* < 0.001), while plant-based protein intake was positively associated with healthier fat sources (*ρ* = 0.46, *p* < 0.001). A positive correlation was found between paying attention to food labels and the consumption of healthier fats (ρ = 0.27, *p* = 0.006). The majority of respondents (75.6%) reported a positive impact of transplantation on eating habits. However, higher post-transplant BMI was associated with less favorable dietary change (OR = 0.94, *p* = 0.018). Longer time since transplantation was also linked to slightly lower likelihood of reporting positive changes (OR = 0.997, *p* = 0.039).

**Conclusion:**

Study results highlight the need to focus on nutritional education in post-transplant care.

## 1 Introduction

Public awareness of the significance of a healthy and balanced diet is growing and it is well-documented that what and how we eat has consequences in several aspects of health – from proper functioning of the digestive tract through better management of chronic diseases to improved overall well-being and mental health (1–3). However, awareness among solid organ transplant recipients regarding appropriate dietary patterns remains insufficient. Adherence to a healthy nutritional regimen may mitigate the risk of many chronic conditions and provide a protective edge, for example against carcinogenesis (4). This is particularly important in patients whose health is already strained.

Solid organ transplantation (Tx) is a life-saving treatment for end-stage organ failure. It is preferred to dialysis in younger patients with kidney insufficiency (5–8). However, life after Tx requires immunosuppressive medications to prevent rejection, and these drugs have numerous metabolic consequences. Glucocorticosteroids may increase appetite and contribute to hypertension, hyperglycaemia and weight gain (9). Calcineurin inhibitors (tacrolimus and cyclosporine) can cause hypertension and dyslipidaemia while mycophenolic acid may lead to haematologic disturbances (10–13). These side effects add-up to the cardiovascular risks already pre-existing in transplant recipients (14). Although the metabolic consequences of transplantation are well established, data on actual post-transplant dietary behaviors remain limited.

Weight gain after transplantation is common. Observational studies have shown that kidney transplant recipients gain an average of 5–10 kg in the first year post-surgery and this gain is associated with hypertension, diabetes, dyslipidaemia and reduced graft survival (15). In a German cohort of 433 transplant patients, obesity prevalence increased from 14.8% before transplantation to 19.9% after, and higher post-transplant BMI was linked to new-onset diabetes and decreased graft function (16). Despite these risks, there are limited nutrition guidelines for transplant recipients and evidence-based dietary counselling is rarely reported in routine care (17). A better understanding of post-transplant dietary behaviors could help clinicians identify patients at risk and tailor educational interventions. Therefore, the present study aims to evaluate dietary behaviors among recipients of different solid organs, providing comparative data that may help identify specific gaps and inform targeted nutritional interventions.

*Research question*: The aim of this cross-sectional study is to characterise self-reported dietary habits among adult solid organ transplant recipients in a single centre in Poland and to explore how these behaviors relate to body-mass index and time since transplantation. By mapping dietary gaps in this population, we hope to identify opportunities for targeted post-transplant care and education.

## 2 Materials and methods

### 2.1 Study group and survey distribution

This was a cross-sectional survey carried out between July and November 2023 at a single transplant centre in Poland, which cares for roughly 2,500 adults with functioning kidney, liver, pancreas, heart or lung grafts. During routine inpatient stays and outpatient visits, trained members of the research team invited patients to complete a paper questionnaire. Outpatients completed the questionnaires in the waiting area of the outpatient clinic, while inpatients completed them during their hospital stay. The questionnaire was designed to assess participants’ usual dietary habits, rather than their intake during the current week or during hospitalization. The average time required to complete the questionnaire was approximately 25 min. No assistance was provided during questionnaire completion. Because we collected data at a single point in time, we recorded only the body-mass index reported during the survey and could not assess pre-transplant weight or changes over time. Participation was voluntary, questionnaires were anonymous and no incentives were offered. Patients gave verbal consent before taking part.

The questionnaire was predominantly frequency based and did not require estimation of exact portion sizes. Most items assessed the habitual frequency of consumption of specific food groups rather than quantitative intake. One item asked participants to indicate whether their fruit and vegetable intake was below the 400 g per day guideline.

Four researchers were responsible for introducing the study, distributing and collecting questionnaires. After data collection, two team members entered responses from the paper forms into an electronic database for analysis.

### 2.2 Construction of the questionnaire

The first part of the questionnaire included demographic characteristics of the respondents (gender, height, weight and year of birth) and patient data such as the type of organ and the month / year of the Tx. No exact dates of Tx were provided to avoid identification of the respondents.

Patients were asked to fill out a table containing 12 questions (Q1–Q12) assessing their eating habits, each addressing different aspects of dietary behavior. Respondents could choose from four frequency options: “every day,” “most days of the week,” “2–3 times a week,” and “once a week or less.”

The questions investigated the consumption of vegetables and fruit, wholegrain products, unsweetened milk, processed meat, sources of fats (animal vs. plants) in the diet and consuming sweetened beverages or fruit juices. The survey also included questions about eating behaviors such as visiting bars or restaurants, consumption in front of electronic screens (without estimating the duration of such a distracted meal), paying attention to the labels of food products, and consuming more meals than before the transplantation.

At the end of the questionnaire, respondents were asked to mark how the transplantation has influenced their dietary habits on a scale from −5 to +5. Answers from −5 to −1 meant that the transplantation had a negative impact, 0 meant no impact, and answers from +1 to +5 meant a positive impact, with −5 representing an extremely negative influence and +5 indicating an extremely positive influence.

The thresholds for ‘adequate’ and ‘inadequate’ intake of each dietary component were based on WHO recommendations - Nutrition for a health and guidelines from the National Centre for Nutrition Education, Poland (56). For example, consumption of ≥400 g of fruits and vegetables per day was considered adequate, while intake below this level was classified as inadequate.

The survey was based on a model applied by Jodczyk et al. following written permission to use the format in our research (18). The full questionnaire is available in Supplementary material.

### 2.3 Statistical analysis

The significance level was set at alpha = 0.05. Continuous variables were expressed as medians (Mdn) with interquartile ranges (IQR). Categorical variables were presented as frequencies and percentages. Group comparisons for numerical variables utilized the Wilcoxon rank sum test, and the Chi- square or Fisher’s exact test for categorical data, depending on sample size.

As the questionnaire comprised items spanning heterogeneous dietary domains rather than a single latent construct, internal consistency assessment via Cronbach’s alpha was considered methodologically inappropriate. The instrument functioned as a multidimensional behavioral frequency inventory, consistent with its original application by Jodczyk et al. (18).

### 2.4 Cumulative link model

The association between multiple predictors and the ordinal outcome was evaluated using a cumulative link model (CLM) for ordinal regression, as described by Chistensen (19). Predictor selection was guided by a stepwise regression approach with backward elimination, utilizing Akaike’s Information Criterion (AIC) to optimize model fit (28).

Model adequacy was rigorously assessed through the Hosmer-Lemeshow goodness-of-fit test (20) while the proportional odds assumption was validated using the Brant test (19). The model’s explanatory capacity, quantified by pseudo-R^2^, achieved a value of 0.31 with square transformation and 0.32 with logarithmic transformation, indicating a satisfactory level of variance explanation in the ordinal response variable.

To account for the non-symmetric distribution of ordinal outcome, cumulative probabilities were modeled with a complementary log–log link function, effectively capturing the skewness inherent in the data. Confidence intervals and *p*-values were estimated using Wald’s z-distribution approximation to ensure robust inference (21).

#### 2.4.1 A multivariate analysis: identification of factors determining the overall impact of transplantation on dietary behavior

A CLM was initially constructed, including three sociodemographic variables (gender, age, BMI), five clinical variables, and twelve survey responses (Q1–Q12). The stepwise regression algorithm with backward elimination reduced the number of predictions from 20 to six, enhancing model fit, as indicated by a reduction in AIC from 508.75 to 468.92. The final model achieved a more parsimonious structure while maintaining explanatory strength.

##### 2.4.1.1 Model specification

The optimized CLM included BMI, time since transplantation and four responses Q1, Q2, Q7, and Q11 as significant predictors. These variables represent both continuous and categorical data, reflecting key clinical and behavioral aspects influencing dietary outcomes post-transplantation.

##### 2.4.1.2 Assessment of model covergence

The model successfully converged after seven iterations, with minimal gradient and negligible log-likelihood error, confirming the optimalization process was effective. Diagnostic tests indicated that the proportional odds assumption was met, as verified by the omnibus test (X^2^(42) = 48.56, *p* = 0.0230) and individual assessment of each predictor. These results confirm the robustness and reliability of the model’s estimation.

##### 2.4.1.3 Model fit and predictive power

Goodness-of-fit was validated using the Hosmer-Lemeshow test (X^2^(32) = 39.34, *p* = 0.174), indicating alignment between predicted and observed outcomes. The model’s explanatory capacity, assessed via R^2^, ranged from 0.31 (square-root transformation) to 0.32 (logarithmic transformation), reflecting adequate variance explanation in the ordinal outcome.

##### 2.4.1.4 Threshold interpretation and predictive robustness

Log-likelihood plots demonstrated clear threshold demarcations between response categories, particularly distinguishing between “negative” to “rather negative” and “rather positive” to “positive.” This precision implies the model effectively captures the gradient of dietary behavior impact based on the analyzed predictors.

### 2.5 Characteristics of the statistical tool

Analyses were conducted using the R Statistical language (version 4.3.3; 42) on Windows 11 pro 64 bit (build 22,631), using the packages *marginaleffects* (version 0.23.0; 43), *ordinal* (version 2023.12.4.1; (19)), *ggeffects* (version 1.5.1; 44), *sjPlot* (version 2.8.15; 45), *parameters* (version 0.22.2; 46), *report* (version 0.5.8; 47), *correlation* (version 0.8.5; 48), *ggstatsplot* (version 0.12.3; 49), *gtsummary* (version 1.7.2; 50), *gofcat* (version 0.1.2; 51), *MASS* (version 7.3.60.0.1; 52), *reshape2* (version 1.4.4; 53), *ggplot2* (version 3.5.0; 54) and *dplyr* (version 1.1.4; 55).

### 2.6 Ethics statement and permissions

According to the Act of 5 December 1996 on the professions of physicians and dentists (Journal of Laws of 2021, item 790 as amended), the presented study was not a medical experiment. The study aligns with the Institutional Ethics Committee and the Helsinki Declaration (1964). The study was approved by the Bioethical Commission, Medical University of Warsaw (number AKBE/227/2023). The Chief of the Department of Transplantation, Nephrology, Immunology and Internal Diseases at the Medical University of Warsaw gave permission to carry out the study.

## 3 Results

### 3.1 Characteristic of the study cohort

**Figure 1:**
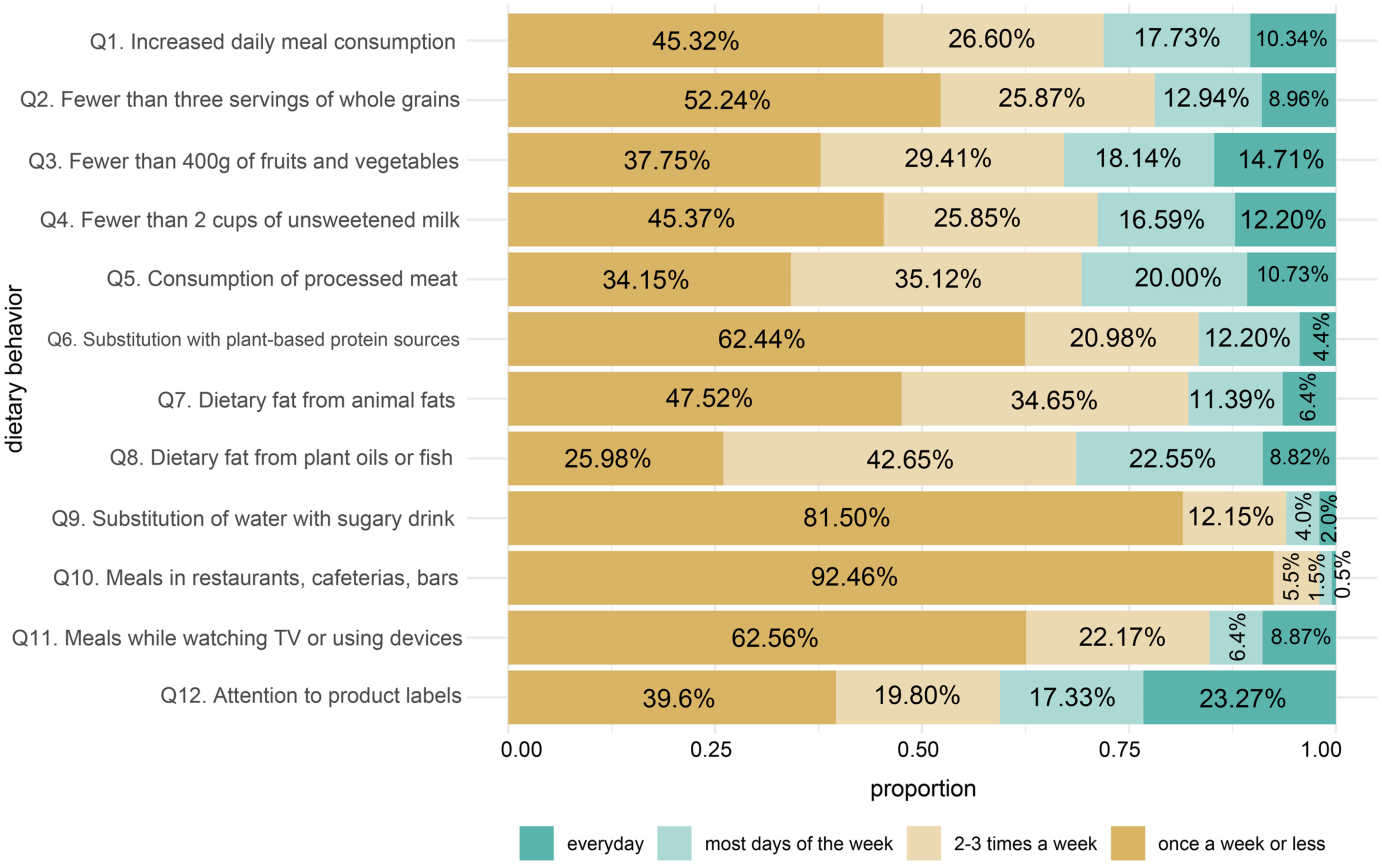
Proportional bar plot of responses to survey items Q1 - Q12. Q1. consuming more meals per day than before transplantation (including snacking); Q2. consuming less than 3 servings of wholegrain products daily; Q3. consuming less than 400 g vegetables and fruits per day; Q4. consuming less than 2 glasses of unsweetened milk or other dairy products daily; Q5. consuming products containing processed meat, such as sausages, ham, frankfurters etc.; Q6. replacing meat by protein rich plant products such as nuts and legumes: beans, chickpeas, soy, lentils, fava bean, peas; Q7. consuming products which are source of animal fats or trans fatty acids present in products, such as pastries, candy bars, salty snacks and fast-food products; Q8. consuming products, which are source of unsaturated fatty acids, such as canola oil, olive oil or fish; Q9. drinking sweetened beverages or fruit juices instead of water; Q10. meals in restaurants, canteens, bars; Q11. consuming meals while looking at the screen of TV, computer or other devices; Q12. paying attention to labels of chosen products during shopping, taking into account ingredients, amount of calories etc.

**Figure 2:**
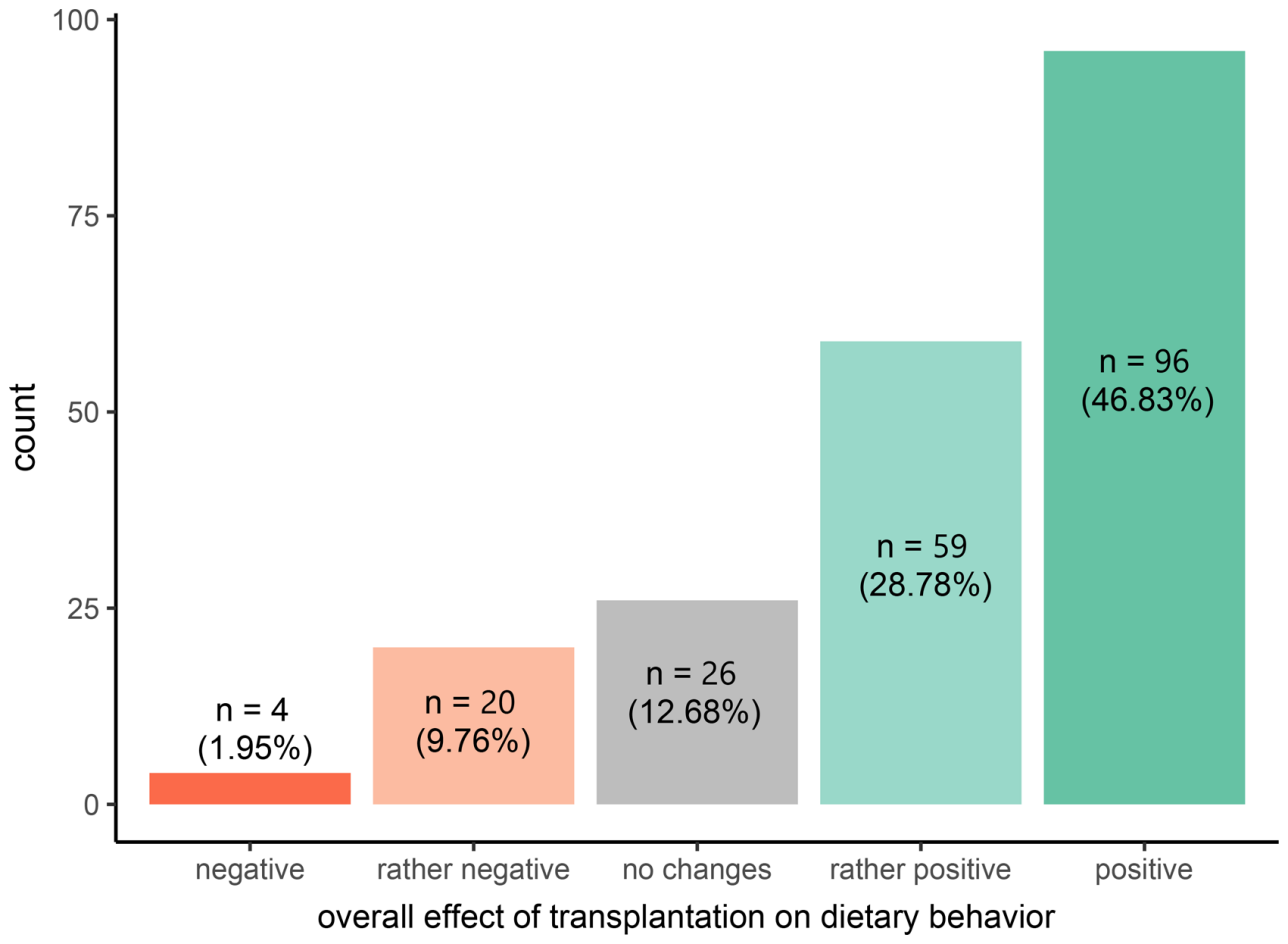
Self-assessment responses of overall effect of transplantation on eating habits and subsequently analyzed in a multivariate framework in this form, *N* = 205 participants.

The initial number of all questionnaires collected for the study was 212. Patients whose survey responses lacked a significant amount of data were excluded from the analysis, resulting in 205 participants, aged 19 to 81 years. Transplant characteristics are provided in Table 1. However, complete data for all questionnaire items regarding nutrition / dietary patterns were available for 192 participants. Therefore, the results presented in Figure 1, Tables 2, 4 were based on 192 participants. Tables 1, 3, and Figure 2, however, included the results of all 205 participants. Of these, 44.9% (*n* = 92) were females and 55.1% (*n* = 113) were males, with a male-to-female ratio of 1.23:1. The median age was 50 years (*IQR*: 38–60). The median BMI was 24.86 kg/m^2^ (*IQR*: 22.28–28.23). By BMI classification, 50.7% (*n* = 104) were of normal weight, 32.7% (*n* = 67) were overweight, 14.6% (*n* = 30) were obese, and 2.0% (*n* = 4) were underweight.

**Table 1:** Transplant characteristics for overall cohort and stratified by gender, *N* = 205.

Characteristic	*N*	Overall cohort^a^	Transplant recipient’s gender		*p* ^c^
			Female, *n* = 92^a^	Male, *n* = 113^a^	
Number of transplants	205				0.118
One		193 (94.15%)	84 (91.30%)	109 (96.46%)	
Two		12 (5.85%)	8 (8.70%)	4 (3.54%)	
Kidney transplant		159 (77.56%)	70 (76.09%)	89 (78.76%)	0.648
Liver transplant		45 (21.95%)	21 (22.83%)	24 (21.24%)	0.785
Pancreas transplant		10 (4.88%)	7 (7.61%)	3 (2.65%)	0.116^d^
Time since last transplantation in months		57.00 (23.00, 136.00)^b^	45.00 (19.00, 119.50)^b^	65.00 (23.00, 138.00)^b^	0.472^e^

a n (%).

b Mdn (IQR).

c Pearson’s Chi-squared test.

d Fisher’s exact test.

e Wilcoxon rank sum test.

**Table 2:** Association of BMI and time since last transplantation with dietary outcomes, *N* = 192.

Predictors	Overall impact of transplantation on dietary behavior		
	OR	CI 95%	p
BMI	0.94	0.89–0.99	**0.018**
Time from last transplantation	0.997	0.99–1.00	**0.039**
Q1. Increased daily meal consumption			
Everyday	Reference level		
Most days a week	0.42	0.14–1.30	0.134
2–3 times a week	0.26	0.09–0.79	**0.017**
Once a week or less	0.18	0.06–0.51	**0.001**
Q2. Fewer than three servings of whole grains			
Everyday	Reference level		
Most days a week	0.36	0.13–0.99	**0.047**
2–3 times a week	0.93	0.36–2.41	0.874
Once a week or less	0.64	0.26–1.55	0.320
Q7. Dietary fat from animal fats			
Everyday	Reference level		
Most days a week	7.61	2.65–21.81	**< 0.001**
2–3 times a week	4.63	1.91–11.23	**< 0.001**
Once a week or less	8.70	3.43–22.08	**< 0.001**
Q11. Meals while watching TV or using devices			
Everyday	Reference level		
Most days a week	0.33	0.10–1.04	0.058
2–3 times a week	0.95	0.36–2.51	0.917
Once a week or less	0.59	0.24–1.44	0.250

OR, odds ratio; CI 95%, confidence interval 95%; p, *p*-value of statistical test. Bold values mean: statistically significant.

**Table 3:** BMI profile of transplant recipients stratified by gender, *N* = 205.

Characteristic	N	Transplant recipient’s gender		*p* ^c^
		Female, *n* = 92^a^	Male, *n* = 113^a^	
Age, years	205	46.50 (37.00, 60.00)	51.00 (39.00, 60.00)	0.385
BMI, kg/m^2^	205	24.05 (21.09, 27.60)	25.46 (23.39, 28.68)	**0.003**
BMI group	205			0.083^d^
Normal		50 (54.35%)^b^	54 (47.79%)^b^	
Obesity		11 (11.96%)*^b^*	19 (16.81%)^b^	
Overweight		27 (29.35%)^b^	40 (35.40%)^b^	
Underweight		4 (4.35%)^b^	0 (0%)^b^	

a Mdn (IQR).

b n (%).

c Wilcoxon rank sum test.

d Fisher’s exact test.

Bold values mean: statistically significant.

**Table 4:** Regression intercept thresholds, *N* = 192.

Intercept	Overall impact of transplantation on dietary behavior		
	OR	CI 95%	*p*
Negative | rather negative	0.00	0.00–0.02	**< 0.001**
Rather negative | no changes	0.01	0.00–0.09	**< 0.001**
No changes | rather positive	0.02	0.00–0.20	**< 0.001**
Rather positive | positive	0.08	0.01–0.67	**0.020**

OR, odds ratio; CI 95%, confidence interval 95%; p, *p*-value of statistical test. Bold values mean: statistically significant.

Based on the results in Table 3 there was no significant age difference between male and female transplant recipients (*p* = 0.385). However, males had a significantly higher median BMI (25.46 kg/m^2^) than females (24.05 kg/m^2^, *p* = 0.003).

Males were more frequently classified as overweight (35.4%) and obese (16.8%), while females were more likely to have a normal BMI (54.4%). A small proportion of females (4.4%) were underweight, with no underweight males. Although the distribution of BMI categories^1^ approached significance (*p* = 0.083), the overall trend suggests males may be at greater risk for weight-related complications post-transplant.

### 3.2 Transplant characteristics

The analysis of transplant characteristics for the overall cohort and stratified by gender presented in Table 1 provides key insights into the distribution of transplant-related variables in the study population. The distribution of transplant characteristics, including the number of transplants, type of organ transplanted, and time since last transplant, is largely comparable between male and female recipients.

The vast majority of patients (94.2%) underwent a single transplant, with a slightly higher proportion of males (96.5%) compared to females (91.3%). Although this difference did not reach statistical significance (*p* = 0.118), the trend suggests that female recipients may undergo multiple transplants more frequently, with 8.7% of females receiving two transplants compared to 3.5% of males.

Kidney transplantation was the most common procedure, accounting for 77.6% of the cohort, with no significant difference between females (76.1%) and males (78.8%) (*p* = 0.648). Similarly, liver transplantation was performed in 22.0% of patients, with comparable rates between females (22.8%) and males (21.2%) (*p* = 0.785). These findings suggest that the type of organ transplanted does not differ significantly by gender in this cohort.

The median time since the last transplant was 57 months for the entire cohort, with no significant difference between females (*Mdn* = 45.00 months) and males (*Mdn* = 65.00 months) (*p* = 0.472).

### 3.3 Characteristics of survey

The post-transplant patient nutrition survey consisted of 12 questions, each addressing various aspects of dietary behavior. The distribution of responses for each question is illustrated in Figure 1.

For participants who underwent two transplants (*n* = 12, 5.9%), time since last transplant refers to the interval from the most recent transplantation procedure to the date of survey completion.

### 3.4 Analysis of correlations between survey items Q1–Q12

The results of correlation analysis were presented as a correlation matrix in Figure A.1. Pairwise Spearman rank correlation coefficients between all 12 dietary behavior items (Q1–Q12), with Holm-adjusted significance annotations, are presented in the Figure A.1.

Specifically, a significant finding is a positive correlation between the consumption of fewer servings of whole grains (Q2) and fewer fruits and vegetables (Q3), with a correlation coefficient of rho = 0.26, *p* = 0.009. This relationship suggests that patients who fail to meet the recommended intake for one food group are also likely to fall short in another. A strong positive correlation is also observed between the consumption of processed meat products (Q5) and dietary fat from animal sources (Q7), with rho = 0.41 and *p* < 0.001. Another significant correlation is found between the use of plant-based protein sources (Q6) and the consumption of dietary fats from plant oils or fish (Q8), showing rho = 0.46 and *p* < 0.001. A positive correlation is also noted between paying attention to product labels (Q12) and the consumption of dietary fats from plant oils or fish (Q8), with rho = 0.27 and *p* = 0.006.

Another significant correlation is found between the use of plant-based protein sources (Q6) and the consumption of dietary fats from plant oils or fish (Q8), showing rho = 0.46 and p < 0.001. The results show that positive dietary behaviors tend to occur together, such as choosing plant-based proteins and healthy fats or reading product labels.

Marginally significant correlations support the idea that also certain negative dietary behaviors may cluster. For example, the relationship between the substitution of water with sugary drinks (Q9) and eating meals while watching TV or using electronic devices (Q11) shows rho = 0.22 and *p* = 0.098. Another marginally significant finding is the negative correlation between higher processed meat consumption (Q5) and lower attention to product labels (Q12), with rho = −0.23 and *p* = 0.056. Similarly, the negative correlation between ingestion of dietary fat from animal sources (Q7) and lower attention to product labels (Q12), with rho = −0.23 and *p* = 0.078, points to a broader trend in which patients who consume less healthy fats may also be less inclined to monitor the nutritional content of their food.

### 3.5 Characteristics of the overall effect of transplantation on dietary behavior

The distribution of self-assessed overall impact of transplantation on dietary habits, preliminarily converted to a five-point ordinal scale (negative: raw score −5 or −4, rather negative: raw score −3, −2, or −1, no changes: score 0, rather positive: raw score 1, 2, or 3, positive: raw score 4 or 5), is presented in Figure 2.

The largest proportion of respondents (46.8%, *n* = 96) reported a “positive” impact, followed by 28.8% (*n* = 59) who indicated a “rather positive” effect. A smaller percentage of participants (12.7%, *n* = 26) noted “no changes” in their eating habits post-transplantation. In contrast, 9.8% (*n* = 20) perceived the effect as “rather negative,” while only a minimal proportion (2.0%, *n* = 4) assessed the impact as “negative.” This distribution highlights a predominantly positive self-assessed influence of transplantation on dietary behavior.

The regression intercept thresholds in Table 4 indicate significant separation between the different levels of the outcome, with all thresholds being significant. This suggests that the model effectively differentiates between categories of the overall impact of transplantation on dietary behavior.

#### 3.5.1 Association of BMI and time since transplantation with overall dietary outcomes

Intercept thresholds represent the boundaries between neighboring categories of the ordinal outcome in the cumulative link model. Each threshold indicates the point on the underlying dietary behavior scale where the probability shifts from one category to the next. Statistically significant thresholds (*p* < 0.05) mean that the model can reliably distinguish between adjacent categories. Detailed results are presented in Table 2.

BMI shows a statistically significant association with the overall impact of transplantation, with an *OR* = 0.94 (*p* = 0.018). This indicates that for each unit increase in BMI, the odds of a more positive impact on dietary behavior decrease by 6%, suggesting that higher BMI is associated with a more negative dietary outcome.

Similarly, the time since the last transplantation has a small but significant effect, with an *OR* = 0.997 (*p* = 0.039). Although the effect size is small, it suggests that as time from transplantation increases, there is a slight decrease in the odds of reporting a positive dietary impact.

For *Q1* (Increased daily meal consumption), the frequency of meal consumption is significantly associated with the overall impact. Compared to the reference group of everyday increased meal consumption, those who consume more meals 2–3 times a week have an *OR* = 0.26 (*p* = 0.017), and those who consume more meals once a week or less have an *OR* = 0.18 (*p* = 0.001). These findings indicate that less frequent increase in meal consumption is associated with a significantly more negative dietary impact. However, meal consumption on most days a week does not show a statistically significant difference in impact from everyday frequency (*OR* = 0.42, *p* = 0.134).

For *Q2* (Fewer than three servings of whole grains), consuming fewer than three servings on most days a week has a significant negative association with dietary behavior comparing to everyday frequency (*OR* = 0.36, *p* = 0.047). However, less frequent whole grain consumption (2–3 times a week or once a week or less) was also negatively associated with the outcome, however without statistical significance.

In Q7 (Dietary fat from animal fats), reduced animal fat consumption is strongly associated with a more positive dietary impact. Compared to everyday consumption, consuming animal fat on most days a week (*OR* = 7.61, *p* < 0.001), 2–3 times a week (*OR* = 4.63, *p* < 0.001), or once a week or less (*OR* = 8.70, *p* < 0.001) is linked to significantly higher odds of a positive dietary impact. This suggests that reducing the intake of animal fats is an indicator of positive dietary behaviors.

For Q11 (Meals while watching TV or using devices), eating meals while distracted most days a week shows a trend toward a negative impact on dietary behavior (*OR* = 0.33, *p* = 0.058), though it is not statistically significant. Less frequent distracted eating (2–3 times a week or once a week or less) does not show significant associations.

## 4 Discussion

The findings of our survey reveal various areas where food intake may be improved among post-transplant patients. We recorded routine consumption of processed meat - every day or most days of the week reported by 31%—with rare substitution of animal-based foods with plant-based products—declared less than once a week by 62% of participants. It is generally recognized that intake of high-energy foods such as meat may be justifiable in the initial phase of convalescence following transplantation, when patients are recovering and may experience cachexia or malnutrition (22, 23). In the longer term, however, foods which are naturally rich in dietary fibre, i.e., plants, need to be promoted in order to prevent subsequent weight gain (4). Our data are cross-sectional and we only collected a measure of BMI once in the survey, so we cannot say if participants had put on weight after transplant. However, as much as almost half of those asked were overweight or obese (50% of men and 41% of women).

Observational data indicate that increased BMI is associated with a greater risk of metabolic syndrome and other post-transplant comorbidities (24, 25). Chakkera et al. (26) stated that weight gain after transplantation may result in deteriorated graft function and cardiovascular disease incidence. Excessive weight is a documented issue in kidney transplant recipients (27–29); Moreover, meta-analyses have shown obese patients have increased rates of acute rejection and delayed graft function compared to normal-weighted patients (30). The results of 34% increase in weight over pre-transplant weights in the study of Nöhre et al. and 48% increase in the study of Tarsitano et al. reflect the extent of the issue (27, 31). Our findings form part of a larger pool of evidence, although we are unable to track weight change in the individuals we surveyed.

Non-alcoholic fatty liver disease is now becoming an increasing primary indication for liver transplantation among the transplant population (32). Causes of obesity are multifactorial in themselves—dietary factors, physical inactivity, disturbed sleep, stress and endocrine imbalance are all causal in nature—but inappropriate dietary advice is likely to play a role in abnormal weight gain in transplant recipients (33). Some have advocated improved education of patients and more formalized nutrition programs (34, 35). Our results, despite being restricted to one centre and based on self-reported practice, are consistent with this call: we identified many patients to still be regularly consuming processed meat and hardly ever adopting plant-based protein or unsaturated fats.

In our cohort, women ate healthier than men. Female participants consumed more unsaturated fats and reported less frequency of sweet drinks or fruit juice. They also more often declared healthy BMI (53% vs. 49%) and were less likely to be obese (16% vs. 11%). However, there are mixed findings in the literature for sex differences in the context of BMI and weight gain. Tarsitano et al. did not detect significant gender difference in BMI distribution in kidney transplant recipients (27), whereas Forte et al. (36) reported female gender as a risk factor for post-transplant weight gain. Hap et al. (37) described comparable weight gain in both sexes within 1 year after renal transplant, though cumulative BMI increase was more frequent in females. Maybe one of the reasons for the difference is that our analysis combined recipients of diverse solid organs, while most studies examine one type of transplant.

Time after transplantation also had a statistically significant effect in some answers. Inadequate consumption of whole-grain foods and excessive consumption of animal and trans fats were more prevalent in patients with over 1 year after transplantation. Similarly, decline in healthy habits have been described by Flattery et al. (38) in a cohort of heart transplant recipients several years after transplantation and in a group of middle-aged kidney transplant recipients who followed the Mediterranean diet less diligently than younger recipients as reported by Vučković et al. (39). These trends are predictive of declining healthy behavior over time and attest to the value of continued education.

By assessing product groups rather than specific food items, we were able to estimate which group of patients was more likely to consume higher amounts of saturated versus unsaturated fatty acids and which group consumed less. Moreover, behavior patterns identified as potentially obesogenic, e.g., distracted eating, consuming saturated fat in excess and not reading product labels, are those that tend to be more prevalent among the participants in our study with higher BMI. Our survey demonstrated that those patients who read the food labels are more likely to select healthier fats, and those selecting plant proteins are likely to ingest unsaturated fats. They do so in accordance with Mediterranean diet habits which have been associated with improved cardiovascular and metabolic health. Review by Cyrino et al. and a study by Tarsitano et al. demonstrate the advantages of Mediterranean and DASH diets in kidney transplant recipients (27, 40). However, some of the barriers to enhanced vegetable and fruit consumption in this population are glucocorticosteroid-associated hunger, low baseline dietary literacy and poor transplant-oriented nutritional education (22, 41). The findings here, supported by current literature, indicate that sub-optimal dietary counselling and low adherence to healthy diet patterns remain persistent challenges.

Because our research was based on self-reporting and did not ask about interaction with nutrition specialists, we cannot assess the impact of professional counselling. Cross-sectional study design also forbids us from drawing conclusions about causality. But recording prevailing dietary habits lays a foundation for intervention in this vulnerable population who underwent a costly and life-altering procedure such as transplantation. Any potential opportunity should be taken to optimise their health status. Nutrition education and behavior changes in the form of lifestyle programs could help in preventing posttransplant weight gain. The lack of formal nutritional guidelines available to transplant patients in Poland highlights the value of studies such as ours. Longitudinal studies in the future should follow weight changes and eating patterns over time and establish whether controlled nutrition education can provide additional benefits.

### 4.1 Limitations

This study has several limitations that should be kept in mind. First, it was conducted in a single transplant centre. Although this centre is one of the largest in Poland and cares for patients from across the country, the results may not fully represent practices in other institutions.

Secondly, the survey captured dietary habits and body-mass index at a single time point. We did not have access to pre-transplant weight so we cannot assess how patients’ BMI changed over time or determine causal relationships between diet and weight. Furthermore, the wide range in time since transplantation (IQR: 23–136 months) introduces heterogeneity in dietary behavior that may reflect different post-transplant stages rather than a stable, homogeneous nutritional pattern. Longitudinal designs stratifying patients by post-transplant phase would be necessary to disentangle these effects.

Thirdly, most respondents were kidney transplant recipients, with smaller numbers of other organ recipients, which limited the ability to analyse differences between organ groups. It should be noted that the study population predominantly consisted of kidney transplant recipients, followed by liver transplant recipients; therefore, the findings primarily apply to these groups, which remain the main focus of our study. Future studies should include larger numbers of liver, heart, pancreas, and lung recipients to explore organ-specific patterns.

A practical challenge was the paper-based nature of the survey. In a small number of cases respondents left incomplete or ambiguous answers. Two team members entered the data manually and any unclear responses were discussed by the authors until consensus was reached on whether to include or exclude the questionnaire.

Finally, all dietary information was self-reported. The questionnaire did not ask whether respondents received dietary counselling, and we were unable to verify reported habits with objective measures. These factors may introduce reporting bias.

The survey relied on self-reported data, and participants were not provided with dietitian guidance regarding portion size estimation. Therefore, responses—particularly to the item assessing fruit and vegetable intake relative to the 400 g per day guideline—may be subject to misclassification. However, as all other items were frequency based rather than quantity based, the potential impact of portion size estimation bias was limited.

In spite of these constraints, the study is useful in describing food habits in Polish transplant recipients and highlighting specific areas where dietetic education could be implemented. Multi-centre, longitudinal studies with accurate anthropometric data and queries regarding lifestyle counselling would strengthen and validate these observations.

## 5 Conclusion

Healthy eating and weight control are vital after organ transplantation. In our survey most patients felt their diet improved after transplantation, yet nearly half were overweight or obese and many reported to eat processed meat and animal fats. Longer time since transplantation was linked to a drift towards less healthy eating habits. Encouraging plant-based proteins and more vegetables, while limiting processed meats, should be underlined in transplant care. Because our data were collected at a single time point and relied on self-reports, we cannot assess changes over time. Nonetheless, the patterns observed support calls for ongoing nutrition education and clearer dietary guidance for transplant recipients.

## Data Availability

The original contributions presented in the study are included in the article/Supplementary material, further inquiries can be directed to the corresponding author.

## Footnotes

1 Patients with missing self-assessments of the overall impact of transplantation or with significant missing data in the survey responses were excluded from the analysis.

## Supplementary material

SUPPLEMENTARY FIGURE A1 Survey.
