# Supplementary material for: Dietary habits among solid organ transplant recipients: results from a single-center study in Poland
Source: Front Nutr. 2026 May 20;13:1685314. doi: 10.3389/fnut.2026.1685314 (PMC13229783; doi:10.3389/fnut.2026.1685314)
Supplement: SUPPLEMENTARY FIGURE A1 — Survey. [file Image_1.pdf]

Spearman Correlation Matrix of Dietary Behaviour Items (Q1−Q12)

Dietary Patterns Among Solid Organ Transplant Recipients (N = 205)

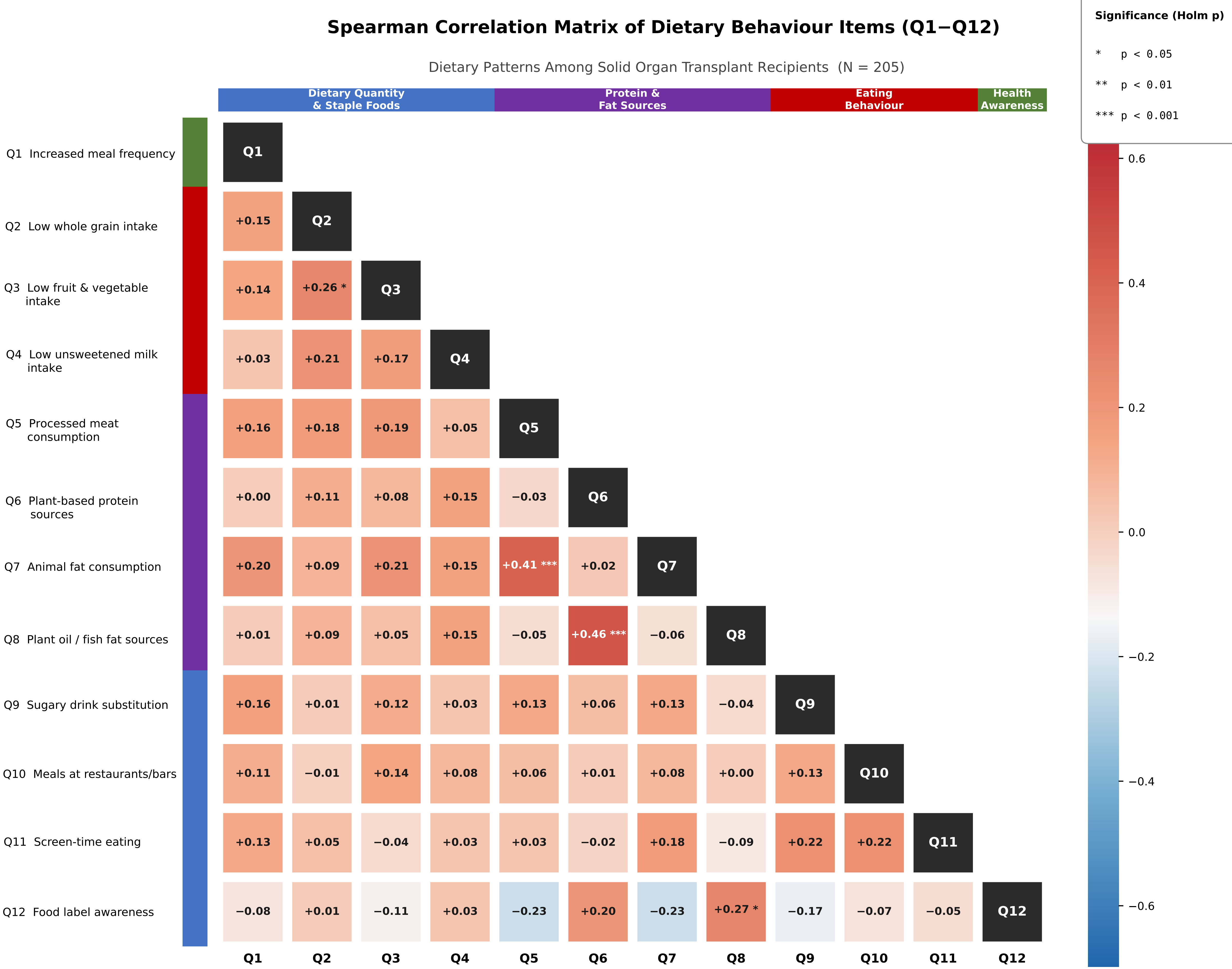

Each cell displays the Spearman rank correlation coefficient ρ between two dietary behaviour items.

*Sign convention: + positive association, − negative association. Superscripts denote statistical significance based on Holm-adjusted p-values: *p < 0.05, **p < 0.01, ***p < 0.001.*

Blue shading: negative correlation; red shading: positive correlation. Colour intensity is proportional to | ρ | (scale:−0.70 to +0.70).

Diagonal cells display item identifiers. Upper triangle is suppressed for readability.Coloured markers on the left axis and header bars denote the thematic domain:

Dietary Quantity & Staple Foods (Q1−Q4), Protein & Fat Sources (Q5−Q8), Eating Behaviour (Q9−Q11), Health Awareness (Q12).
