# Supplementary material for: Dietary habits among solid organ transplant recipients: results from a single-center study in Poland
Source: Front Nutr. 2026 May 20;13:1685314. doi: 10.3389/fnut.2026.1685314 (PMC13229783; doi:10.3389/fnut.2026.1685314)
Supplement: Supplementary file 2 [file Supplementary_file_1.pdf]

Questionnaire

Eating Habits of Patients After Solid Organ Transplantation. Survey Study.

ENGLISH Version

PART 1 - Identification and transplantation questions

1. Gender:
    a. Female
    b. Male
    c. I would rather not to give it

2. Height (in cm): ……………………………………………….

3. Weight (in kg): …………………………………………………..

4. Year of birth: ………………………………………………….

5. Please indicate the organ that has been transplanted and is currently under transplant control.
    a. Kidney
    b. Liver
    c. Heart
    d. Lung
    e. Pancreas
    f. Other

6. If you chose "other" please indicate which organ was involved in the transplant.

    ……………………………………………………………………………………

7. Please provide the month and year of your last transplant.

    ………………………………………………………………………………………

PART 2 - Diet questions

The following questions concern nutrition.
It consists of 12 single-choice questions and one in which you are asked to rate the impact of the transplant on a scale of -5 to +5.

1. How often during a typical week do you have the following situation:

	once a week or less often	2-3 times per week	most days of the week	everyday
consuming more meals per day than before transplantation (including snacking)				
consuming less than 3 servings of wholegrain products daily (less than 90g/day)				
consuming less than 400g vegetables and fruits				
consuming less than 2 glasses of unsweetened milk or other dairy products daily				
consuming products containing processed meat, such as sausages, ham, frankfurters etc.				
replacing meat by protein rich plant products such as nuts and legumes: beans, chickpeas, soy, lentils, fava bean, peas				

	once a week or less often	2-3 times per week	most days of the week	everyday
consuming products which are source of animal fats or trans fatty acids present in products, such as pastries, candy bars, salty snacks and fast-food products				
consuming products, which are source of unsaturated fatty acids, such as canola oil, olive oil or fish				
drinking sweetened beverages or fruit juices instead of water				
meals are taken in restaurants, canteens, bars				
consuming meals while looking at the screen of TV, computer or other devices				
paying attention to labels of chosen products during shopping, taking into account ingredients, amount of calories etc.				

2. How would you rate the overall impact of transplantation on your nutrition compared to before transplantation? Values of -5 to -1 indicate a negative impact and +1 to +5 a positive impact, taking into account their severity; 0 indicates no impact.

   Please circle the appropriate number, where -5 means transplantation had an extremely negative impact; 0 is transplantation had no impact and 5 is transplantation had an extremely positive impact.

-5	-4	-3	-2	-1	0	1	2	3	4	5
